# Machine Learning Models for Slope Stability Classification of Circular Mode Failure: An Updated Database and Automated Machine Learning (AutoML) Approach

**DOI:** 10.3390/s22239166

**Published:** 2022-11-25

**Authors:** Junwei Ma, Sheng Jiang, Zhiyang Liu, Zhiyuan Ren, Dongze Lei, Chunhai Tan, Haixiang Guo

**Affiliations:** 1 Badong National Observation and Research Station of Geohazards (BNORSG), China University of Geosciences, Wuhan 430074, China; 2 Three Gorges Research Center for Geo-Hazards of the Ministry of Education, China University of Geosciences, Wuhan 430074, China; 3 School of Economics and Management, China University of Geosciences, Wuhan 430074, China

**Keywords:** automated machine learning (AutoML), slope stability classification, circular mode failure, hyperparameter tuning, stacked ensemble

## Abstract

Slope failures lead to large casualties and catastrophic societal and economic consequences, thus potentially threatening access to sustainable development. Slope stability assessment, offering potential long-term benefits for sustainable development, remains a challenge for the practitioner and researcher. In this study, for the first time, an automated machine learning (AutoML) approach was proposed for model development and slope stability assessments of circular mode failure. An updated database with 627 cases consisting of the unit weight, cohesion, and friction angle of the slope materials; slope angle and height; pore pressure ratio; and corresponding stability status has been established. The stacked ensemble of the best 1000 models was automatically selected as the top model from 8208 trained models using the H2O-AutoML platform, which requires little expert knowledge or manual tuning. The top-performing model outperformed the traditional manually tuned and metaheuristic-optimized models, with an area under the receiver operating characteristic curve (AUC) of 0.970 and accuracy (ACC) of 0.904 based on the testing dataset and achieving a maximum lift of 2.1. The results clearly indicate that AutoML can provide an effective automated solution for machine learning (ML) model development and slope stability classification of circular mode failure based on extensive combinations of algorithm selection and hyperparameter tuning (CASHs), thereby reducing human efforts in model development. The proposed AutoML approach has the potential for short-term severity mitigation of geohazard and achieving long-term sustainable development goals.

## 1. Introduction

Natural hazards like landslide and subsidence have been acknowledged as a major factor disturbing sustainable development in developing countries [1,2,3,4]. For example, a catastrophic landfill slope failure occurred on 20 December 2015, in Guangming, Shenzhen, China, took the lives of 69 people [5]. The risk assessment and management of natural hazard will have a short-term benefit for severity mitigation and a long-term benefit for achieving sustainable development goals [1].

The evaluation of slope stability is of primary importance for natural hazard risk assessment and management in mountain areas. Numerous efforts have been made for slope stability assessment [6,7,8,9]. However, slope stability assessment for circular mode failure, a typical problem, still remains a challenge for the practitioner and researcher due to inherent complexity and uncertainty [10]. An extensive body of literature exists regarding slope stability assessments of circular failure, and significant progress has been achieved. Three main categories of assessment approaches have emerged: analytical approaches, numerical approaches, and machine learning (ML)-based approaches [11,12,13]. Limited equilibrium methods, such as the simplified Bishop, Spencer, and Morgenstern-Price methods, are commonly used analytical approaches and have been routinely used in practice. Generally, geometrical data, physical and shear strength parameters (unit weight, cohesion, and friction angle), and the pore pressure ratio are required in limited equilibrium methods [14,15]. However, the results vary across different methods due to different assumptions [9]. Numerical approaches (e.g., finite element methods) have been widely adopted for slope stability assessment. However, due to the requirement of numerous expensive input parameters, these models can be applied only in limited cases [16]. Recently, ML-based approaches have led to giant strides in slope stability assessment. A summary of the slope stability assessments of circular failure using ML approaches is given in Table 1. Among the various ML approaches used, artificial neural networks (ANNs) are widely utilized for slope stability assessment due to their simple structure and acceptable accuracy [11,17,18]. Recently, sophisticated ML algorithms, including but not limited to support vector machine (SVM), decision tree (DT), extreme learning machine (ELM), random forest (RF), and gradient boosting machine (GBM) algorithms, have been utilized for slope stability assessment. Hyperparameter tuning is a fundamental step required for accurate ML modeling [19,20]. As listed in Table 1, grid search (GS) and metaheuristic methods, such as the artificial bee colony (ABC) algorithm, genetic algorithm (GA), and particle swarm optimization (PSO), have been utilized for hyperparameter tuning in ML-based slope stability assessment. For example, Qi and Tang [16] simultaneously trained six firefly algorithm (FA)-optimized ML models, including multilayer perceptron neural network, logistic regression (LR), DT, RF, SVM, and GBM models, based on 148 cases of circular mode failure. The FA-optimized SVM was selected as the final model, with an area under the receiver operating characteristic curve (AUC) of 0.967 for the testing dataset. The performance of eight ensemble learning approaches was compared by [12] based on a dataset with 444 cases of circular mode failure. A stacked model was selected as the final model, with an AUC of 0.9452 for the testing dataset.

Although ML-based models have been widely applied, some studies have been based on a small number of samples, which may affect the generalization ability of the classifier. Moreover, most ML models have been manually developed by researchers with expert knowledge in a trial-and-error approach. In fact, exhaustive steps, including data preprocessing [31], feature engineering [32], ML algorithm selection [33], and hyperparameter tuning, are involved in practical applications of ML. Among them, model selection and hyperparameter tuning remain challenges for successful ML-based modeling [34]. Based on the no-free-lunch theorem [35], there is no algorithm that outperforms all others in all problems. Therefore, at present, according to prior experience, candidate off-the-shelf models are trained with a training dataset and validated by researchers. The ML model that provides the best performance is considered the final model and tested with an out-of-box testing dataset. This traditional workflow makes the model development process knowledge-based and time-consuming [36], and might yield unsatisfactory results [37]. However, most practitioners and researchers lack the knowledge and expertise required to build satisfactory ML models. Hence, an objective workflow with less human effort is needed, providing a basis for the concept of automated ML (AutoML) [38].

From the perspective of automation, AutoML is a systematic framework that automates algorithm selection and hyperparameter tuning and explores different combinations of factors with minimal human intervention [34,39,40,41]. AutoML has been successfully applied for ML modeling in a variety of fields, including tunnel displacement prediction [36], tunnel boring machine performance prediction [34], and earthquake casualty and economic loss prediction [42]. Thus, the generalization ability of this approach has been confirmed.

In the present study, an updated database with 627 cases consisting of the unit weight, cohesion, and friction angle of the slope materials: slope angle and height, pore pressure ratio, and corresponding stability status of circular mode failure, has been collected. For the first time, an AutoML approach was proposed for slope stability classification. The top model was selected from 8208 trained ML models by exploring numerous combinations of algorithm selection and hyperparameter tuning (CASHs) with minimal human intervention.

The major contribution of this paper is highlighted as follows:

(a) A large database consisting of 627 cases has been collected for slope stability classification.
(b) Based on the updated dataset, an AutoML approach was proposed for slope stability classification without the need for manual trial and error. The proposed AutoML approach outperformed the existing ML models by achieving superior performance.

The rest of this paper is organized as follows: the updated database and methodology are presented in Section 2 and Section 3, respectively. Section 4 presents and discusses experimental results. Finally, the conclusions and further work are presented in Section 5.

## 2. Database

**Figure 1:**
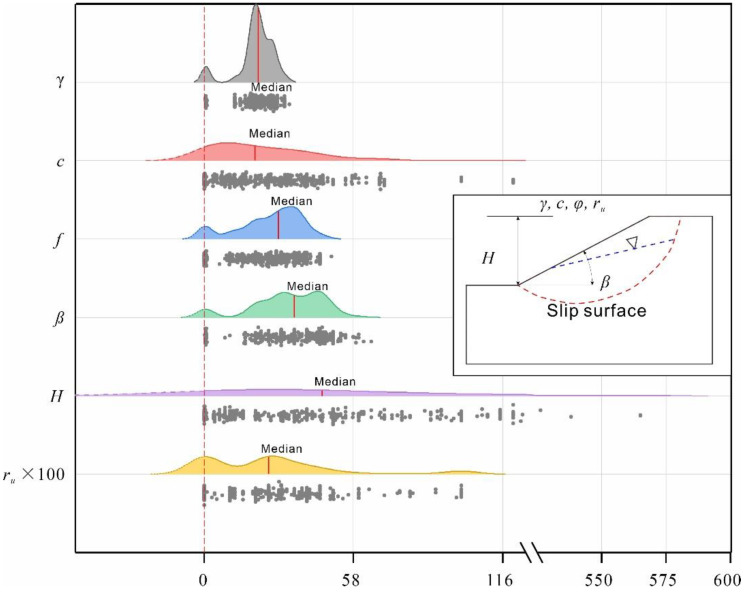
Ridgeline plots showing the density distributions of the input features. The inset shows a schematic diagram of the circular failure model.

As listed in Table 1, the input features relevant to the slope stability assessment of the circular failure model (schematic illustrated in inset of Figure 1) mainly include the unit weight, cohesion, and friction angle of the slope materials, the slope angle and height, and the pore pressure ratio. Moreover, these features are fundamental input parameters for limit equilibrium methods, such as the simplified Bishop method [15,43]. Based on the previous research listed in Table 1, an updated database consisting of 627 cases was obtained from previous studies [11,12,16,24,30,44] and is listed in Appendix A. The database consists of the unit weight, cohesion, and friction angle of the slope materials, the slope angle and height, the pore pressure ratio, and the corresponding stability status. The numbers of positive (stable) and negative (failure) samples are 311 and 316, respectively. The statistics of the input features are summarized in Table 2. To better visualize the collected dataset, ridgeline plots showing the density distributions of the input features based on kernel density estimation [3] are presented in Figure 1. As shown, the collected dataset was distributed in a wide range of regions, and the distribution was not symmetric.

**Figure 2:**
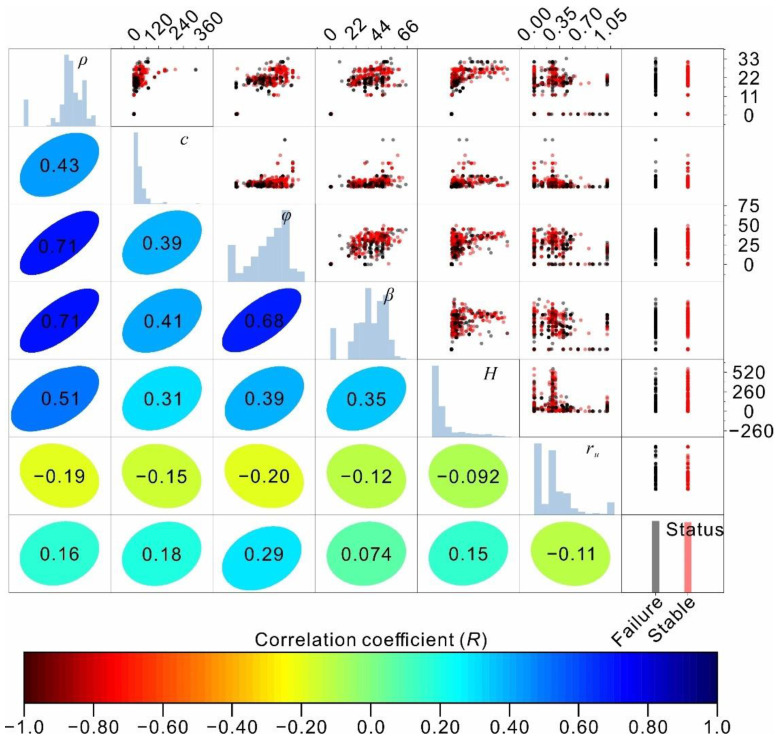
Scatter matrix showing the collected dataset. The panels in the upper right show the data points, and the lower left half of the figure shows the correlation coefficients between the features and the slope stability status.

The Pearson correlation coefficient (R) was adopted to further reveal the linear correlations between input features and the slope stability status and is shown in the lower left half of the panels in Figure 2. As shown, relatively poor linear correlations with correlation coefficients lower than 0.5 were observed between the input features and the slope stability status. Significant linear correlations (R = 0.71, 0.71, and 0.68) were noted for the unit weight, friction angle, and slope angle. Additionally, a moderate correlation (R = 0.51) was found between the unit weight and slope height.

**Figure 3:**
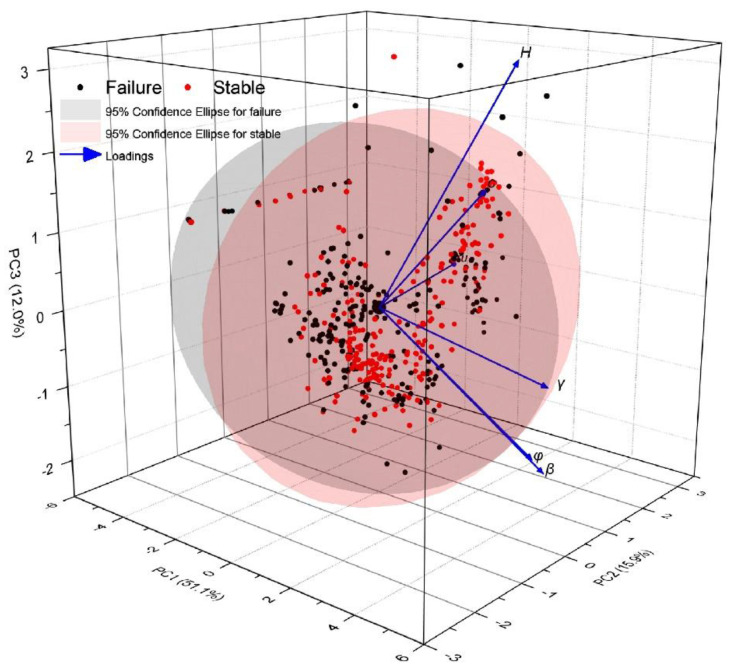
3D PCA score plot of the input features.

Furthermore, the multivariate principal component analysis (PCA) technique [45] was applied to enhance the visualization of the statistical relationships among features. The PCA results shown in Figure 3 demonstrate that the first three principal components (PC1-PC3) account for 79.09% of the entire multivariate variance in space. PC1 is mainly associated with the unit weight, friction angle, and slope angle. PC2 corresponds to the pore pressure ratio. Moreover, overlapping among failure and stability classes can be clearly observed. In other words, the decision boundary for separating slope failure and stability is highly nonlinear and complex.

## 3. Methodology

### 3.1. AutoML

From the perspective of automation, AutoML is a systematic model that automates the algorithm selection and hyperparameter tuning processes and explores different CASHs with minimal human intervention [34,39,40]. More formally, the CASH problem can be stated as follows. Let $A=\{A^{1},A^{2},\cdots ,A^{R}\}$ be a set of ML algorithms, $\Lambda =\{\Lambda^{1},\Lambda^{2},\cdots ,\Lambda^{R}\}$ be the corresponding hyperparameters, and L be the loss function. When adopting k-fold cross validation (CV), the training dataset $D_{training}$ is divided into subsets $\left\{D_{training}^{\left(1\right)},D_{training}^{\left(2\right)},\cdots ,D_{training}^{\left(k\right)}\right\}$ and $\left\{D_{validation}^{\left(1\right)},D_{validation}^{\left(2\right)},\cdots ,D_{validation}^{\left(k\right)}\right\}$. The CASH problem is defined as

(1) $$A^{*}{}_{\lambda^{*}}\in \underset{A^{\left(j\right)}\in A,\lambda^{\left(j\right)}\in \Lambda }{\arg\min}\frac{1}{k}\sum_{i=1}^{k}L(A_{\lambda }^{\left(j\right)},D_{training}^{\left(i\right)},D_{validation}^{\left(i\right)})$$

**Figure 4:**
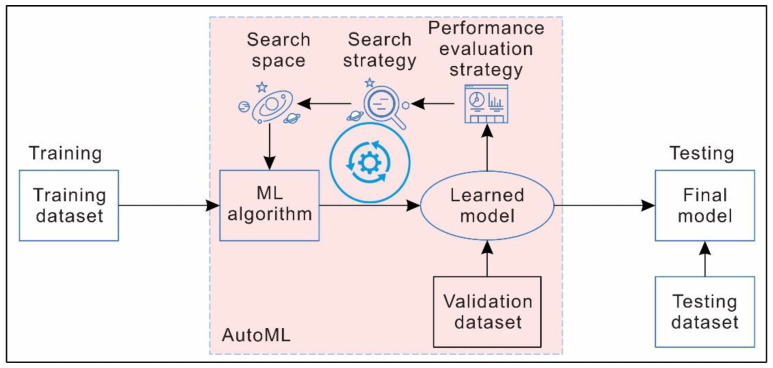
Schematic diagram showing the workflow of AutoML.

Generally, AutoML consists of the following three key components: a search space, a search strategy, and a performance evaluation strategy [40] (schematically illustrated in Figure 4). The search space refers to a set of hyperparameters and the range of each hyperparameter. The search strategy refers to the strategy of selecting the optimal hyperparameters from the search space. Grid search and Bayesian optimization are commonly used search strategies. The performance evaluation strategy refers to the method used to evaluate the performance of the trained models.

Various open-source platforms, such as AutoKeras, AutoPyTorch, AutoSklearn, AutoGluon, and H2O AutoML, have been developed to facilitate the adoption of AutoML [46]. Previous studies [47,48] have demonstrated the strong feature of H2O AutoML for processing large and complicated datasets by quickly searching the optimal model without the need for manual trial and error. Moreover, H2O AutoML provides a user interface for non-experts to import and split datasets, identify the response column, and automatically train and tune models. Therefore, in the present study, the H2O AutoML platform was adopted for the automated assessment of slope.

The H2O AutoML platform includes the following commonly used ML algorithms: generalized linear model (GLM), distributed random forest (DRF), extremely randomized tree (XRT), deep neural network (DNN), and GBM algorithms [49]. The abovementioned ML algorithms in the H2O AutoML platform are briefly described as follows.

GLM is an extended form of a linear model. Given the input variable *x*, the conditional probability of the output class falling within the class *c* of observations is defined as follows:

(2) $${\hat{y}}_{c}=Pr(y=c|x)=\frac{ex^{T}\beta_{c}+\beta_{c0}}{\sum_{k=1}^{K}\left(ex^{T}\beta_{k}+\beta_{k0}\right)}$$

where $\beta_{c}$ is the vector of coefficients for class *c.*

The DRF is an ensemble learning approach based on decision trees. In the DRF training process, multiple decision trees are built. To reduce the variance, the final prediction was obtained by aggregating the outputs from all decision trees.

Similar to the DRF, XRT is based on multiple decision trees, but randomization is strongly emphasized to reduce the variance with little influence on the bias. The following main innovations are involved in the XRT process: random division of split nodes using cut points and full adoption of the entire training dataset instead of a bootstrap sample for the growth of trees.

The DNN in H2O AutoML is based on a multilayer feedforward artificial neural network with multiple hidden layers. There are a large number of hyperparameters involved in DNN training, which makes it notoriously difficult to manually tune. Cartesian and random grid searches are available in H2O AutoML for DNN hyperparameter optimization.

GBM is an ensemble learning method. The basic idea of GBM is to combine weak base learners (usually decision trees) for the generation of strong learners. The objective is to minimize the error in the objective function through an iterative process using gradient descent.

In addition, stacked ensembles can be built using either the best-performing models or all the trained models.

### 3.2. Search Space and Search Strategy

**Figure 5:**
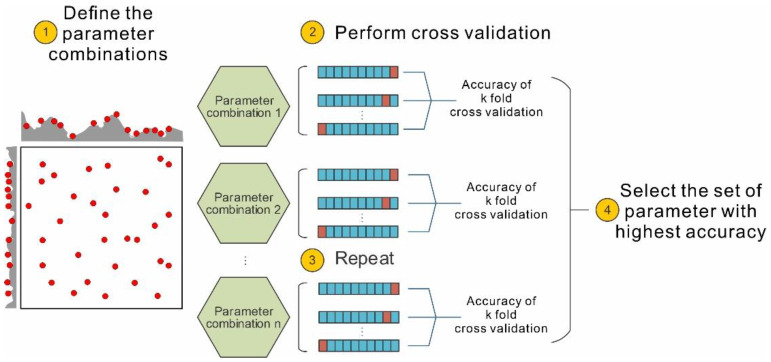
Schematic diagram showing hyperparameter tuning based on the k-fold CV and random grid search methods.

In the present study, a random grid search was adopted for hyperparameter tuning in the search space. When adopting k-fold CV, the hyperparameter tuning process can be described as follows (schematically illustrated in Figure 5). First, possible combinations of the tuned parameters are generated. Then, CV is performed using a possible parameter combination. The training dataset is divided into k equal-sized subsets. A single subset is treated as the validation subset, while the remaining subsets are adopted for classification training. The average accuracy from k validation sets is computed and adopted as the performance measure of the k-CV classifier model. The above process is repeated for all possible parameter combinations. A ranking of all trained classifiers by model performance is obtained. The classifier that yields the highest accuracy is selected.

### 3.3. Performance Evaluation Measures

In the present study, widely applied criteria, including the accuracy (ACC), AUC, sensitivity (SEN), specificity (SPE), positive predictive value (PPV), negative predictive value (NPV), and Matthews correlation coefficient (MCC), were adopted for performance evaluation (Table 3). The AUC can be interpreted as follows: an AUC equal to 1.0 indicates perfect discriminative ability, an AUC value from 0.9 to 1.0 indicates highly accurate discriminative ability, an AUC value from 0.7 to 0.9 indicates moderately accurate discriminative ability, an AUC value from 0.5 to 0.7 demonstrates inaccurate discriminative ability, and an AUC less than 0.5 indicates no discriminative ability.

### 3.4. Slope Stability Assessment through AutoML

**Figure 6:**
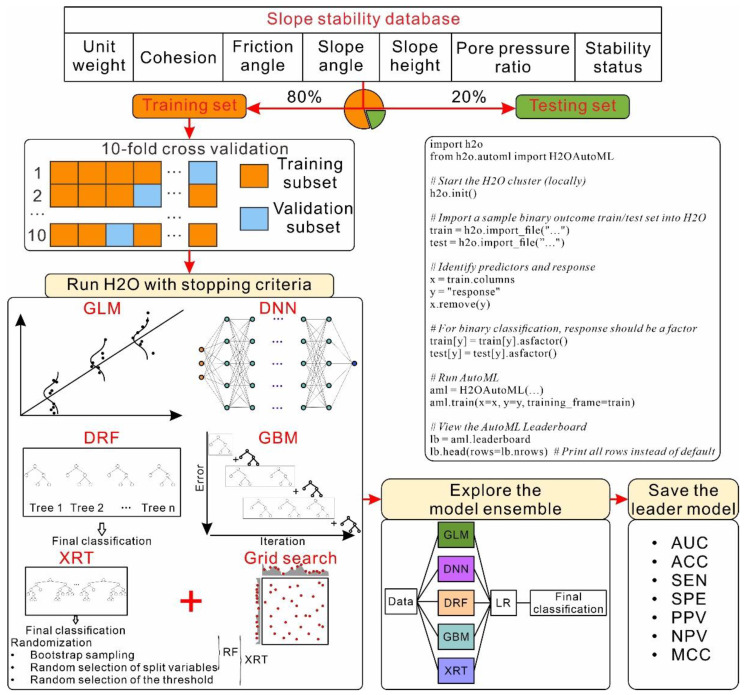
Flowchart of the AutoML-based slope stability classification.

In the present study, the H2O AutoML approach was adopted for ML model development for slope stability classification (schematic illustrated in Figure 6). First, the database listed in Appendix A was randomly divided into training and testing datasets at a ratio of 80% to 20%, respectively. ML models, including GLM, DRF, XRT, DNN, and GBM were automated and developed (schematic illustrated in Figure 6). To enhance the reliability and performance, the common 10-fold CV was performed. A full list of tuned hyperparameters and the corresponding searchable values are given in Table 4. Stacked ensembles were developed based on the best-performing models and all the tuned models. A leaderboard ranking the mode performance accuracy was achieved. The leader models were saved and evaluated on the testing dataset.

The AutoML process was implemented using H2O AutoML (3.36.1.2) with an Intel(R) Xeon(R) E-2176M @ 2.70 GHz CPU with 64 GB RAM. The maximum time allotted to run generation classifiers, except for the stacked ensembles, was set to 3600 s.

## 4. Results and Discussions

### 4.1. Performance Analysis

A total of 8208 ML models, including bypass CV models, were trained with the H2O AutoML platform and saved. The top five models from the leaderboard were selected and listed in Table 4 for testing. The performance evaluation metrics for the top five models on the testing dataset are listed in Table 5.

**Figure 7:**
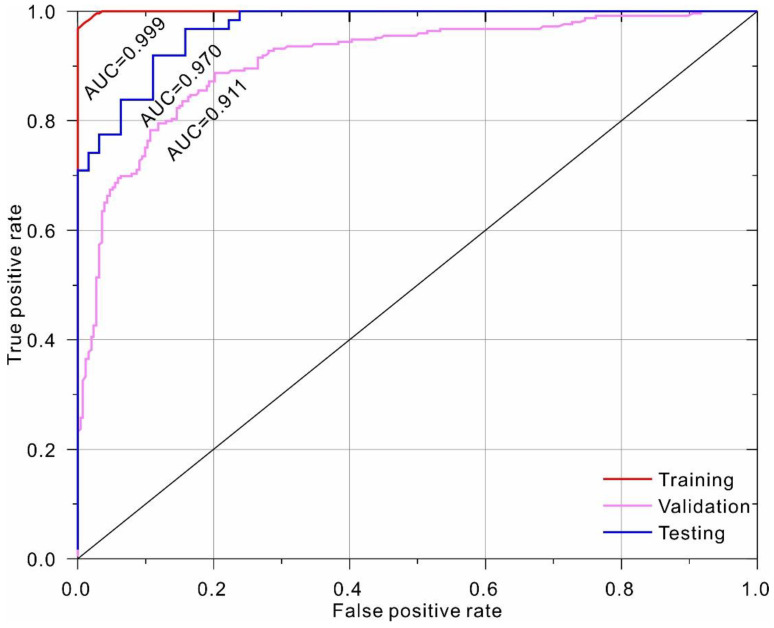
ROC curve of the top-performing model (H2O_1_) from AutoML.

**Figure 8:**
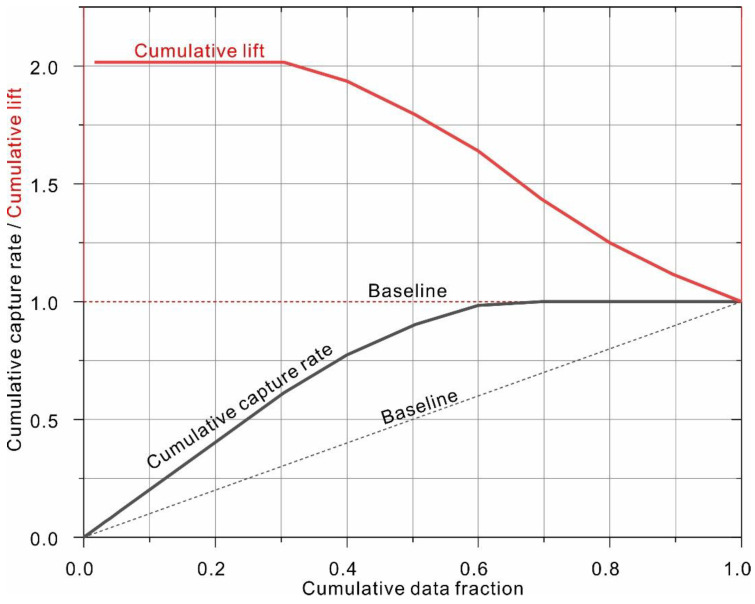
Cumulative gain and lift charts for the top-performing model (H2O_1_) based on testing data.

As listed in Table 5, the stacked ensemble of the best 1000 models (H2O_1_) ranked as the top-performing model. The corresponding ROC curves are shown in Figure 7, which clearly indicates that the top-performing model is capable of providing highly accurate discriminative ability, with AUC of 0.999 and 0.970 for the training and testing dataset, respectively. The model performance was further evaluated using gain and lift charts (Figure 8). A gain chart measures the effectiveness of a classifier based on the percentage of correct classifications obtained with the model versus the percentage of correct classifications obtained by chance (i.e., the baseline). As shown, for the top model, only 30% of the population is required to achieve an accuracy of 60%, compared to 30% for the random model. The top classifier is capable of achieving a maximum lift of 2.1. In other words, when only 10% of the sample was selected, the average accuracy of the top model was approximately two times higher than that of the random model.

**Figure 9:**
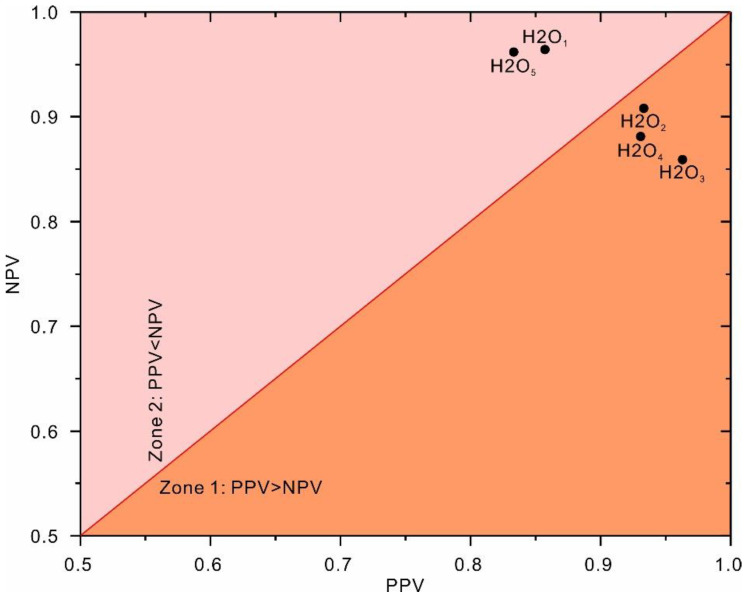
Correlation between the NPV and PPV values of the classification models based on the testing dataset.

Figure 9 demonstrates the correlation between NPV and PPV for the obtained top-five classification models based on the testing dataset. As shown, the top-performing model (H2O_1_) falls within zone 2, in which the obtained NPV is greater than the PPV. This result indicates that the top-performing model (H2O_1_) tends to classify slope status as a failure (negative status) more often than stable (positive status). In other words, the top-performing model (H2O_1_) may overestimate stability.

### 4.2. Model Interpretation

**Figure 10:**
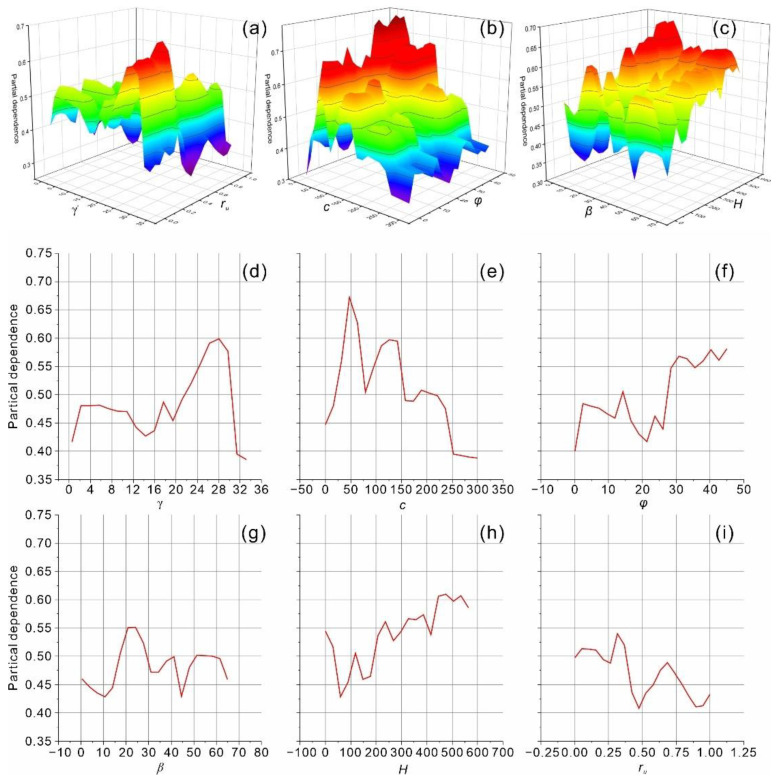
Partial dependence plots of the input features in the top-performing model (H2O_1_) for the classification of slope stability. (**a**) Unit weight and pore pressure ratio, (**b**) cohesion and friction angle, (**c**) slope angle and slope height, (**d**) unit weight, (**e**) cohesion, (**f**) friction angle, (**g**) slope angle, (**h**) slope height, and (**i**) pore pressure ratio.

In the present study, the partial dependence plot graphically revealing the input–output relationship was adopted for model interpretation. The partial dependence plot has been considered as one of the most popular model agnostic tools due to the advantages of simple definition and easy implementation. The partial dependence relations of the input features in the top-performing model (H2O_1_) are shown in Figure 10. In partial dependence plots, features with greater variability have more significant effects on the model [18,50]. As shown, the top-performing model (H2O_1_) is highly influenced by the slope height and friction angle.

### 4.3. Validation of the AutoML Model in ACADS Example

**Figure 11:**
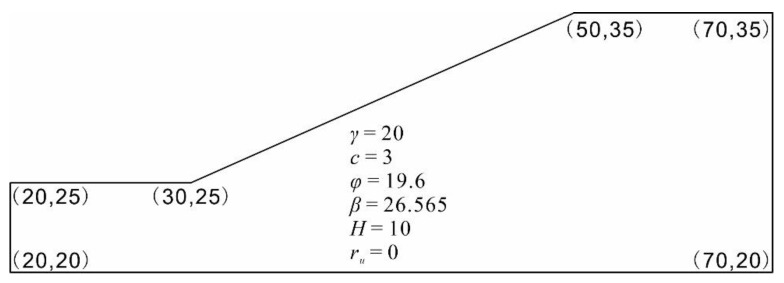
ACADS reference slope example EX1 (Unit: m).

Furthermore, the predictive capacity of the top-performing model (H2O_1_) was validated on the Australian Association for Computer-Aided Design (ACADS) referenced slope example EX1, which is a simple homogeneous slope. The slope is 20 m long and 10 m high. The geometry and material properties are shown in Figure 11. With the parameters listed in Figure 11, the example slope was estimated to fail [43]. The top-performing model (H2O_1_) successfully classified the slope example as a failure case.

### 4.4. Comparison with Exiting Models

To further assess performance, the top-performing model (H2O_1_) from the AutoML approach was further compared with a manually derived ML model for slope stability assessment (Table 6). As shown in Table 6, in the previous studies, the firefly algorithm optimized SVM (FA-SVM) provides the best performance with an AUC of 0.967 [16], followed by ensemble classifiers on the extreme gradient boosting (XGB-CM) [11]. Obviously, the top-performing model (H2O_1_) is of better generalization ability than the existing models shown in Table 6 with the largest AUC and ACC values. These comparative results clearly indicate that the top-performing model (H2O_1_) from AutoML approach is capable of providing better generalization performance than the manually derived ML and metaheuristics-optimized model.

### 4.5. Advantages and Limitations of the Proposed Approach

Generally, the traditional ML models require workflows which encompass data preprocessing, feature engineering, ML algorithm selection, and hyperparameter tuning to be constructed, and are often developed based on prior experience. Due to varying levels of knowledge, the traditional ML model may not fully exploit the power of ML, resulting in less optimal results than those obtained with other models. Therefore, it is not objective to claim that one algorithm outperforms another without adjusting the hyperparameters. In contrast, AutoML is capable of automatically implementing the above processes and extensively exploring different workflows with minimal human intervention, resulting in a better model. In fact, previous studies [51,52] have reported that AutoML outperformed traditional ML models that were manually developed by data scientists. Moreover, it takes less computational time to train AutoML, with hundreds of optional pipelines, than it does to train a manually derived ML model, often requiring days to tune. In fact, based on the collected dataset, the computational time of AutoML with 8408 pipelines is one hour. Moreover, various commercial and open-source AutoML platforms have been developed, and many successful implementations have been reported. For example, an AutoML vision model was implemented for production recommendation using Google Cloud AutoML without hiring ML engineers [40]. These results may suggest that AutoML is preferred in some cases. However, due to the complex and involved process required to build an AutoML system from scratch, AutoML is still in an early stage of development. At present, AutoML is not fully automated [37,40]. For example, human efforts are still needed for data collection and data cleaning. For now, clear objectives based on high-quality data must be defined for AutoML. Nevertheless, the AutoML approach holds limitations such as black box, and is computationally expensive for large-scale datasets due to extensive searching of different pipelines.

## 5. Conclusions

In the present study, an updated database consisting of 627 cases was collected for slope stability classification of circular failure model. For the first time, an AutoML approach was proposed for ML model development. Instead of manually building a pipeline for ML algorithm selection and hyperparameter tuning, AutoML is capable of automatically implementing model development and performing extensive searches of different pipelines with minimal human intervention. The stacked ensemble of the best 1000 models was selected as the top model from 8208 ML trained models. The top-performing model provided highly accurate discriminative ability, with an AUC of 0.970 and an ACC of 0.904 for the testing dataset, achieving a maximum lift of 2.1. The trained AutoML model outperformed traditional manually tuned and metaheuristic-optimized models. AutoML was verified as an effective tool for automated ML model development and slope stability assessments of circular failure.

Given the successful use of AutoML for classification of slope stability for circular mode failure, it seems that such a methodology could be useful for short-term severity mitigation of geohazard and achieving long-term sustainable development goals.

Although the proposed AutoML approach shows promising results, it still has some limitations. Beyond the black box nature, among the major shortcomings of AutoML, a solution is their computational complexity. Future works should focus on developing explainable and interpretable ML models by coupling data-driven models with physical models.

## Figures and Tables

**Table 1:** Summary of the slope stability assessment of circular mode failure using MLs.

Reference	Data Size (Stable/Failure)	Input Features	Data Preprocessing	ML Algorithm Selection	Hyperparameter Tuning	Final Model and Performance
[21]	82 (38/44)	γ, c, φ, β, H, $\;r_{u}$	/	BP	Trial and error GA	GA-optimized BP was selected as the final model, with an AUC of 0.455 for the testing dataset.
[22]	32 (14/18)	γ, c, φ, β, H, $\;r_{u}$	/	ANN	Trial and error	The ANN achieved an ACC of 1.00 for the testing dataset in two cases.
[23]	46 (17/29)	γ, c, φ, β, H, $\;r_{u}$	Data normalization	SVM	PSO	PSO-SVM achieved an ACC of 0.8125 for the testing dataset.
[24]	168 (84/84)	γ, c, φ, β, H, $\;r_{u}$	Data normalization	LSSVM	FA	The FA-optimized LSSVM achieved an AUC of 0.86 for the testing dataset.
[25]	168 (84/84)	γ, c, φ, β, H, $\;r_{u}$	Data normalization	RBF LSSVM ELM	Orthogonal least squares GA Trial and error	The GA-ELM was selected as the final model, with an AUC of 0.8706 for the testing dataset.
[26]	82 (49/33)	γ, c, φ, β, H, $\;r_{u}$	/	NB	/	NB achieved an ACC of 0.846 for the testing dataset.
[27]	107 (48/59)	γ, c, φ, β, H, $\;r_{u}$	/	RF SVM Bayes GSA	Ten-fold CV	The GSA was selected as the final model, with an AUC of 0.889 for the testing dataset.
[17]	168 (84/84)	γ, c, φ, β, H, $\;r_{u}$	Data normalization	GP QDA SVM ADB-DT ANN KNN Classifier ensemble	GA	The optimum ensemble classifier was selected as the final model, with an AUC of 0.943 for the testing dataset.
[16]	148 (78/70)	γ, c, φ, β, H, $\;r_{u}$	Data normalization	LR DT RF GBM SVM BP	FA GS	The FA-optimized SVM was selected as the final model, with an AUC of 0.967 for the testing dataset.
[18]	221 (115/106)	γ, c, φ, β, H, $\;r_{u}$	Data normalization	ANN SVM RF GBM	Five-fold CV	The GBM-based model was selected as the final model, with an AUC of 0.900 for the testing dataset.
[28]	87 (42/45)	γ, c, φ, β, H, $\;r_{u}$	/	J48	Trial and error	J48 achieved an ACC of 0.9231 for the testing dataset.
[13]	257 (123/134)	γ, c, φ, β, H, $\;r_{u}$	/	XGB RF LR SVM BC LDA KNN DT MLP GNB XRT Stacked ensemble	ABC PSO	The stacked ensemble was selected as the final model, with an AUC of 0.904 for the testing dataset.
[11]	153 (83/70)	γ, c, φ, β, H, $\;r_{u}$	Data normalization and outlier removing	KNN SVM SGD GP QDA GNB DT ANN Bagging ensemble Heterogeneous ensemble	GS	An ensemble classifier based on extreme gradient boosting was selected as the final model, with an AUC of 0.914 for the testing dataset.
[29]	19 (13/6)	γ, c, φ, β, H, $\;r_{u}$	Data normalization	K-means cluster	HS	K-means clustering optimized by HS achieved an ACC of 0.89 for all datasets.
[12]	444 (224/220)	γ, c, φ, β, H, $\;r_{u}$	Data normalization	AdaBoost GBM Bagging XRT RF HGB Voting Stacked	GS	A stacked model was selected as the final model, with an AUC of 0.9452 for the testing dataset.
[30]	422 (226/196)	γ, c, φ, β, H, $\;r_{u}$	Data normalization	MDMSE	GS	The MDMSE model achieved an AUC of 0.8810 for the testing dataset.

Note: Abbreviations in this table are explained in Abbreviations.

**Table 2:** Summary of the input feature statistics.

Input Feature	Notation	Range	Median	Mean	Std.
Unit weight (kN/m^3^)	γ	0.492–33.160	20.959	20.185	7.044
Cohesion (kPa)	c	0–300.00	19.690	25.600	31.036
Friction angle (°)	φ	0–49.500	28.800	25.308	12.331
Slope angle (°)	β	0.302–65.000	34.980	32.605	13.711
Slope height (m)	H	0.018–565.000	45.800	90.289	120.140
Pore pressure ratio	$r_{u}$	0–1.000	0.250	0.254	0.260

**Table 3:** Confusion matrix and performance measures for slope stability assessment.

	Predicted	Stable	Failure	
Actual				
**Stable**		True positive (TP)	False negative (FN)	Sensitivity: $SEN=\frac{TP}{TP+FN}$ (The ideal value is 1, whereas the worst is zero.)
**Failure**		False positive (FP)	True negative (TN)	Specificity $SPE=\frac{TN}{FP+TN}$ (The ideal value is 1, whereas the worst is zero.)
		Positive predictive value (PPV) $PPV=\frac{TP}{TP+FP}$ (The ideal value is 1, whereas the worst is zero.)	Negative predictive value (NPV) $NPV=\frac{TN}{FN+TN}$ (The ideal value is 1, whereas the worst is zero.)	Accuracy $ACC=\frac{TP+TN}{TP+FN+FP+TN}$ (The ideal value is 1, whereas the worst is zero.) Matthews correlation coefficient $MCC=\frac{TP\cdot TN-FP\cdot FN}{\sqrt{\left(TP+FP)\cdot (TP+FN)\cdot (TN+FP)\cdot (TN+FN\right)}}$ (The ideal value is 1.)

**Table 4:** The hyperparameter search space for GS optimization for AutoML-based slope stability classification.

Algorithm	Parameter	Searchable values
DNN	Adaptive learning rate time smoothing factor (epsilon)	$\left\{10^{-6},10^{-7},10^{-8},10^{-9}\right\}$
	Hidden layer size (hidden)	Grid search 1: {20}, {50}, {100}
		Grid search 2: {20, 20}, {50, 50}, {100, 100}
		Grid search 3: {20, 20, 20}, {50, 50, 50}, {100, 100, 100}
	Hidden_dropout_ratio	Grid search 1: {0.1}, {0.2}, {0.3}, {0.4}, {0.5}
		Grid search 2: {0.1, 0.1}, {0.2, 0.2}, {0.3, 0.3}, {0.4, 0.4}, {0.5, 0.5}
		Grid search 3: {0.1, 0.1, 0.1}, {0.2, 0.2, 0.2} {0.3, 0.3, 0.3}, {0.4, 0.4, 0.4}, {0.5, 0.5, 0.5}
	Input_dropout_ratio	{0.0, 0.05, 0.1, 0.15, 0.2}
	Adaptive learning rate time decay factor (rho)	{0.9, 0.95, 0.99}
GLM	Regularization distribution between L1 and L2 (alpha)	{0.0, 0.2, 0.4, 0.6, 0.8, 1.0}
GBM	Column sampling rate (col_sample_rate)	{0.4, 0.7, 1.0}
	Column sample rate per tree (col_sample_rate_per_tree)	{0.4, 0.7, 1.0}
	Maximum tree depth (max_depth)	{3, 4, 5, 6, 7, 8, 9, 10, 11, 12, 13, 14, 15, 16, 17}
	Minimum number of observations for a leaf (min_rows)	{1, 5, 10, 15, 30, 100}
	Minimum relative improvement in squared error reduction (min_split_improvement)	$\left\{10^{-4},10^{-5}\right\}$
	Row sampling rate (sample_rate)	{0.50, 0.60, 0.70, 0.80, 0.90, 1.00}

**Table 5:** Comparison of the performance of the selected top-five models from AutoML in slope stability assessments of circular mode failure based on the selected test data.

Model ID	Model Type	Hyperparameters	AUC	Confusion Matrix				Performance Measures
H2O_1_	Stacked ensemble	The base models are the top-1000 trained models, and the metalearner is a GLM. A logit transformation is used for the predicted probabilities.	0.970		Predicted	Stable	Failure	SEN = 0.968 SPE = 0.841 PPV = 0.857 NPV = 0.964 ACC = 0.904 MCC = 0.815
				Actual				
				Stable		60	2	
				Failure		10	53	
H2O_2_	GBM	score_tree_interval = 5; ntrees = 105; max_depth = 7; stopping_metric = logloss; stopping_tolerance = 0.045; learn_rate = 0.1; learn_rate_annealing = 1; sample_rate = 1; col_sample_rate = 0.4; col_sample_rate_change_per_level = 1; col_sample_rate_per_tree = 0.7	0.968		Predicted	Stable	Failure	SEN = 0.903 SPE = 0.937 PPV = 0.933 NPV = 0.908 ACC = 0.920 MCC = 0.840
				Actual				
				Stable		56	6	
				Failure		4	59	
H2O_3_	DRF	Ntrees = 50; max_depth = 20	0.963		Predicted	Stable	Failure	SEN = 0.839 SPE = 0.968 PPV = 0.963 NPV = 0.859 ACC = 0.904 MCC = 0.815
				Actual				
				Stable		52	10	
				Failure		2	61	
H2O_4_	XR	score_tree_interval = 5; max_after_balance_size = 5; max_confusion_matrix_size = 20; ntrees = 50; max_depth = 20; stopping_metric = logloss; stopping_tolerance = 0.045; sample_rate = 0.632	0.963		Predicted	Stable	Failure	SEN = 0.871 SPE = 0.937 PPV = 0.931 NPV = 0.881 ACC = 0.904 MCC = 0.810
				Actual				
				Stable		54	8	
				Failure		4	59	
H2O_5_	GBM	score_tree_interval = 5; ntrees = 97; max_depth = 7; stopping_metric = logloss; stopping_tolerance = 0.045; learn_rate = 0.1; learn_rate_annealing = 1; sample_rate = 0.8; col_sample_rate = 0.8; col_sample_rate_change_per_level = 1; col_sample_rate_per_tree = 0.8	0.960		Predicted	Stable	Failure	SEN = 0.968 SPE = 0.810 PPV = 0.833 NPV = 0.962 ACC = 0.888 MCC = 0.786
				Actual				
				Stable		60	2	
				Failure		12	51	

**Table 6:** Comparison of different ML models for slope stability assessments of circular mode failure.

Reference	Model	AUC	ACC	Reference	Model	AUC	ACC
[24]	BDA LM-ANN SCG-ANN RMV SVM RBP-ANN MO-LSSVM	0.75 0.79 0.81 0.83 0.83 0.84 0.86	/	[25]	RBF LSSVM ELM	*/*	0.81 0.8706 0.8400
[17]	GA-GP GA-QDA GA-SVM GA-ANN GA-ADB-DT GA-KNN GA-OEC	0.893 0.798 0.908 0.877 0.936 0.908 0.943	/	[27]	RF SVM NB GSA	0.833 0.556 0.667 0.886	/
[16]	FA-LR FA-DT FA-MLP FA-RF FA-GBM FA-SVM	0.822 0.854 0.864 0.957 0.962 0.967	/	[18]	ANN SVM RF GBM	0.888 0.889 0.897 0.900	/
[13]	XGB RF LR SVM BC LDA KNN DT MLP GNB XRT Stacked ensemble	0.77 0.79 0.83 081 0.71 0.80 0.78 0.72 0.83 0.7. 0.74 0.90	/	[11]	KNN SVM SGD GP QDA GNB DT ANN B-KNN B-SVM B-ANN RF AB GBM XGB Heterogeneous ensemble	0.931 0.796 0.688 0.933 0.817 0.775 0.829 0.817 0.938 0.892 0.933 0.904 0.910 0.929 0.950 0.950	0.839 0.806 0.710 0.839 0.774 0.806 0.774 0.806 0.871 0.871 0.839 0.806 0.839 0.774 0.903 0.806
[12]	GBM Bagging Adaboost XRT RF HGB Voting Stacked	0.9199 0.9291 0.9199 0.9519 0.9268 0.8970 0.9588 0.9382	/	[30]	SVM DT LR NB Boosting MDMSE	/	0.8452 0.8333 <0.75 <0.75 0.8214 0.8810
Current study	H2O_1_ (Stacked Ensemble_Best1000)	***0.970***	***0.904***				

Note: The best results are shown in bold italics. The results for relatively small sample sets (less than 100) are not presented or compared.

## Data Availability

The data used are contained in Appendix A.

## Abbreviations

AB: adaptive boost; ABC: artificial bee colony; ACC: accuracy; ACADS: Australian Association for Computer Aided Design; ADB: adaptive boosted decision tree; ANN: artificial neural network; AUC: area under the receiver operating characteristic curve; AutoML: automated machine learning; B-ANN: bagging artificial neural network; BC: bagging classifier; BDA: Bayes discriminant analysis; B-KNN: bagging k-nearest neighbors; BP: back-propagation; B-SVM: bagging support vector machine; CASHs: combinations of algorithm selection and hyperparameter tuning; CV: cross validation; DNN: deep neural network; DRF: distributed random forest; DT: decision tree; ELM: extreme learning machine; FA: firefly algorithm; GA: genetic algorithm; GBM: gradient boosting machine; GLM: generalized linear model; GNB: Gaussian naive bayes; GP: Gaussian process; GS: grid search; GSA: gravitational search algorithm; HGB: hist gradient boosting classifier; HS: harmony search, KNN: k-nearest neighbors; LDA: linear discriminant analysis; LM: Levenberg–Marquardt; LR: logistic regression; LSSVM: least squares support vector machine; MDMSE: margin distance minimization selective ensemble; ML: machine learning; MLP: multilayer perceptron; MO: metaheuristic optimized; NB: naive Bayes; NPV: negative predictive value; OEC: optimum ensemble classifier; PC: principal component; PCA: principal component analysis; PPV: positive predictive value; PSO: particle swarm optimization; QDA: quadratic discriminant analysis; RBF: radial basis function; RBP: resilient back-propagation; RF: random forest; RMV: relevance vector machine; SCG: scaled conjugate gradient; SEN: sensitivity; SGD: stochastic gradient descent; SPE: specificity; Std.: standard deviation; SVM: support vector machine; XRT: extremely randomized tree.

## Appendix A. Updated Dataset for Slope Stability Assessments of Circular Mode Failure

**Table:**

No	γ **(kN/m^3^)**	c (kPa)	φ(°)	β (°)	H (m)	$r_{u}$	Status
1	17.98	4.95	30.02	19.98	8	0.3	Stable
2	18	5	30	20	8	0.3	Stable
3	21.47	6.9	30.02	31.01	76.8	0.38	Failure
4	21.51	6.94	30	31	76.81	0.38	Failure
5	21.78	8.55	32	27.98	12.8	0.49	Failure
6	21.82	8.62	32	28	12.8	0.49	Failure
7	22.4	10	35	30	10	0	Stable
8	21.4	10	30.34	30	20	0	Stable
9	22.4	10	35	45	10	0.4	Failure
10	27.3	10	39	41	511	0.25	Stable
11	27.3	10	39	40	470	0.25	Stable
12	22.4	10	35	30	10	0.25	Stable
13	21.4	10	30.34	30	20	0.25	Stable
14	27	10	39	41	511	0.25	Stable
15	27	10	39	40	470	0.25	Stable
16	27.3	10	39	40	480	0.25	Stable
17	21.36	10.05	30.33	30	20	0	Stable
18	19.97	10.05	28.98	34.03	6	0.3	Stable
19	22.38	10.05	35.01	30	10	0	Stable
20	22.38	10.05	35.01	45	10	0.4	Failure
21	19.08	10.05	9.99	25.02	50	0.4	Failure
22	19.08	10.05	19.98	30	50	0.4	Failure
23	18.83	10.35	21.29	34.03	37	0.3	Failure
24	16.5	11.49	0	30	3.66	0	Failure
25	16.47	11.55	0	30	3.6	0	Failure
26	19.03	11.7	27.99	34.98	21	0.11	Failure
27	19.06	11.7	28	35	21	0.11	Failure
28	19.06	11.71	28	35	21	0.11	Failure
29	19.06	11.75	28	35	21	0.11	Failure
30	14	11.97	26	30	88	0	Failure
31	19.63	11.97	20	22	12.19	0.41	Failure
32	14	11.97	26	30	88	0.45	Failure
33	19.63	11.97	20	22	21.19	0.4	Failure
34	18.5	12	0	30	6	0	Failure
35	18.5	12	0	30	6	0.25	Failure
36	19.6	12	19.98	22	12.2	0.41	Failure
37	13.97	12	26.01	30	88	0	Failure
38	18.46	12	0	30	6	0	Failure
39	13.97	12	26.01	30	88	0.45	Failure
40	27.3	14	31	41	110	0.25	Stable
41	27	14	31	41	110	0.25	Stable
42	18.84	14.36	25	20	30.5	0	Stable
43	18.84	14.36	25	20	30.5	0.45	Failure
44	18.84	14.36	25	20.3	50	0.45	Failure
45	18.8	14.4	25.02	19.98	30.6	0	Stable
46	18.8	14.4	25.02	19.98	30.6	0.45	Failure
47	18.8	15.31	30.02	25.02	10.6	0.38	Stable
48	18.84	15.32	30	25	10.67	0.38	Stable
49	20.56	16.21	26.51	30	40	0	Failure
50	20.6	16.28	26.5	30	40	0	Failure
51	27.3	16.8	28	50	90.5	0.25	Stable
52	27	16.8	28	50	90.5	0.25	Stable
53	20.96	19.96	40.01	40.02	12	0	Stable
54	21.98	19.96	36	45	50	0	Failure
55	19.97	19.96	36	45	50	0.25	Failure
56	19.97	19.96	36	45	50	0.5	Failure
57	18.77	19.96	9.99	25.02	50	0.3	Failure
58	18.77	19.96	19.98	30	50	0.3	Failure
59	21.98	19.96	22.01	19.98	180	0	Failure
60	21.98	19.96	22.01	19.98	180	0.1	Failure
61	22	20	36	45	50	0	Failure
62	20	20	36	45	50	0.25	Failure
63	20	20	36	45	50	0.5	Failure
64	18	24	30.15	45	20	0.12	Failure
65	17.98	24.01	30.15	45	20	0.12	Failure
66	18.83	24.76	21.29	29.2	37	0.5	Failure
67	20.41	24.9	13	22	10.67	0.35	Stable
68	20.39	24.91	13.01	22	10.6	0.35	Stable
69	18.5	25	0	30	6	0	Failure
70	18.5	25	0	30	6	0.25	Failure
71	18.46	25.06	0	30	6	0	Failure
72	18.77	25.06	19.98	30	50	0.2	Failure
73	18.77	25.06	9.99	25.02	50	0.2	Failure
74	27.3	26	31	50	92	0.25	Stable
75	27	26	31	50	92	0.25	Stable
76	18.68	26.34	15	35	8.23	0	Failure
77	18.66	26.41	14.99	34.98	8.2	0	Failure
78	28.4	29.41	35.01	34.98	100	0	Stable
79	28.44	29.42	35	35	100	0	Stable
80	18.77	30.01	9.99	25.02	50	0.1	Stable
81	18.77	30.01	19.98	30	50	0.1	Stable
82	20.96	30.01	35.01	40.02	12	0.4	Stable
83	18.97	30.01	35.01	34.98	11	0.2	Stable
84	27.3	31.5	29.7	41	135	0.25	Stable
85	27	31.5	29.7	41	135	0.25	Stable
86	27	32	33	42.6	301	0.25	Failure
87	27	32	33	42.4	289	0.25	Stable
88	27	32	33	42	289	0.25	Stable
89	20.39	33.46	10.98	16.01	45.8	0.2	Failure
90	20.41	33.52	11	16	45.72	0.2	Failure
91	20.41	33.52	11	16	45.7	0.2	Failure
92	20.96	34.96	27.99	40.02	12	0.5	Stable
93	27	35	35	42	359	0.25	Stable
94	27	37.5	35	37.8	320	0.25	Stable
95	27	37.5	35	38	320	0.25	Stable
96	28.4	39.16	37.98	34.98	100	0	Stable
97	28.44	39.23	38	35	100	0	Stable
98	27	40	35	43	420	0.25	Failure
99	19.97	40.06	30.02	30	15	0.3	Stable
100	19.97	40.06	40.01	40.02	10	0.2	Stable
101	20.96	45.02	25.02	49.03	12	0.3	Stable
102	17.98	45.02	25.02	25.02	14	0.3	Stable
103	25	46	35	47	443	0.25	Stable
104	25	46	35	44	435	0.25	Stable
105	25	46	35	46	432	0.25	Stable
106	25	46	35	46	393	0.25	Stable
107	25	48	40	49	330	0.25	Stable
108	26.43	50	26.6	40	92.2	0.15	Stable
109	26.7	50	26.6	50	170	0.25	Stable
110	27	50	40	42	407	0.25	Stable
111	25	55	36	45.5	299	0.25	Stable
112	25	55	36	44	299	0.25	Stable
113	18.84	57.46	20	20	30.5	0	Stable
114	18.8	57.47	19.98	19.98	30.6	0	Stable
115	26.8	60	28.8	59	108	0.25	Stable
116	31.3	68	37	47	213	0.25	Failure
117	31.3	68	37	46	366	0.25	Stable
118	31.3	68.6	37	47	305	0.25	Failure
119	16	70	20	40	115	0	Failure
120	15.99	70.07	19.98	40.02	115	0	Failure
121	22.38	99.93	45	45	15	0.25	Stable
122	22.4	100	45	45	15	0.25	Stable
123	25	120	45	53	120	0	Stable
124	24.96	120.04	45	53	120	0	Stable
125	26.49	150	33	45	73	0.15	Stable
126	26.7	150	33	50	130	0.25	Stable
127	26.89	150	33	52	120	0.25	Stable
128	26	150	45	30	200	0.25	Stable
129	26	150.05	45	50	200	0	Stable
130	25.96	150.05	45	49.98	200	0	Stable
131	26.81	200	35	58	138	0.25	Stable
132	26.57	300	38.7	45.3	80	0.15	Failure
133	26.78	300	38.7	54	155	0.25	Failure
134	19.9652	19.95665	36	44.997	50	0.25	Failure
135	25.6	38.8	36	25	26	0	Stable
136	22.88	0	31.78	36.86	45.45	0.54	Failure
137	23.5	25	20	49.1	115	0.41	Stable
138	16	7	20	40	115	0	Failure
139	27.3	37.3	31	30	30	0	Stable
140	22	0	36	45	50	0.25	Stable
141	27	31.5	30	41	135	0.25	Stable
142	18.8008	14.4048	25.02	19.981	30.6	0	Stable
143	19.6	17.8	29.2	46.8	201.2	0.37	Stable
144	18.84	15.32	30	25	10.7	0.38	Stable
145	25	46	36	44.5	299	0.25	Stable
146	19.63	11.98	20	22	12.19	0	Failure
147	25	12	45	53	120	0	Stable
148	18.7724	30.01	9.99	25.016	50	0.1	Stable
149	25	46	35	47	443	0.29	Stable
150	18.7724	25.05835	9.99	25.016	50	0.2	Failure
151	30.95	30.79	27.08	39.77	131.22	0.22	Stable
152	17.4	14.95	21.2	45	15	0.4	Failure
153	23.1	25.2	29.2	36.5	61.9	0.4	Stable
154	21.51	6.94	30	31	76.8	0.38	Failure
155	20.9592	45.015	25.02	49.025	12	0.3	Stable
156	27	32	33	42.6	301	0.29	Failure
157	15.9892	70.07335	19.98	40.015	115	0	Failure
158	12	0	30	45	8	0.29	Failure
159	25	46	35	50	285	0.25	Stable
160	13.9728	12.004	26.01	29.998	88	0.45	Failure
161	18.68	26.34	15	35	8.23	0.25	Failure
162	18.7724	30.01	19.98	29.998	50	0.1	Stable
163	22	0	40	33	8	0.35	Stable
164	20	0	36	45	50	0.25	Failure
165	31.3	68.6	37	47	305	0	Failure
166	22	10	35	45	10	0.403	Failure
167	18	5	26.5	15.52	53	0.4	Failure
168	21.7	32	27	45	60	0	Failure
169	14	11.97	26	30	88	0.25	Failure
170	18.84	14.36	25	20	30.5	0.25	Stable
171	12	0	30	45	4	0.25	Stable
172	18	5	22	15.52	53	0.4	Failure
173	26.2	44.14	32.26	37.71	359.04	0.21	Stable
174	19.9652	19.95665	36	44.997	50	0.5	Failure
175	22	20	36	45	50	0.25	Failure
176	12	0	30	35	4	0	Stable
177	25	120	45	53	120	0.25	Stable
178	31.3	68	37	46	366	0	Failure
179	26.5	36.1	31	35	39	0	Stable
180	20.9592	30.01	35.01	40.015	12	0.4	Stable
181	27.3	10	39	40	470	0.29	Stable
182	27.3	36	1	50	92	0.29	Stable
183	18.84	0	20	20	7.62	0.45	Failure
184	26.2	41.5	36	35	30	0	Stable
185	27.4	38.1	31	25	42	0	Stable
186	26.93	0	41.13	31.68	8.16	0.3	Stable
187	20.8	15.6	20	30	45	0	Failure
188	27	27.3	29.1	34	126.5	0.3	Failure
189	30.33	15.62	24.21	52.5	85.76	0.25	Failure
190	19	11.9	20.4	21.04	54	0.75	Stable
191	18.8	9.8	21	19.29	39	0.25	Failure
192	21.1	34.2	26	30	75	0	Failure
193	20	0.1	36	45	50	0.29	Failure
194	24	0	40	33	8	0.3	Failure
195	24.45	11.34	39.31	44.03	9.79	0.43	Failure
196	18	0	30	33	8	0.303	Stable
197	20.41	24.91	13	22	10.67	0.35	Stable
198	21.8	31.2	25	30	60	0	Failure
199	20	0.1	36	45	50	0.503	Failure
200	24	0	40	33	8	0.303	Stable
201	26.78	26.79	30.66	43.66	249.7	0.25	Stable
202	31.25	25.73	27.97	48.23	91.55	0.21	Failure
203	12	0.03	30	35	4	0.29	Failure
204	22	0	36	45	50	0.25	Failure
205	25	55	36	45	239	0.25	Stable
206	23	24	19.8	23	380	0	Failure
207	21.2	0	35	23.75	150	0.25	Failure
208	20.9592	34.96165	27.99	40.015	12	0.5	Stable
209	12	0	30	45	8	0.25	Failure
210	27	70	22.8	45	60	0.32	Stable
211	18.7724	19.95665	19.98	29.998	50	0.3	Failure
212	28.44	29.42	35	35	100	0.25	Stable
213	20.8	15.4	21	30	53	0	Failure
214	19.596	12.004	19.98	21.995	12.2	0.405	Failure
215	22.1	24.2	39.7	45.8	49.5	0.21	Stable
216	22.4	29.3	26	50	50	0	Failure
217	20	0	24.5	20	8	0.35	Stable
218	25	55	36	45.5	299	0	Stable
219	17.55	22.08	0	34.99	5.88	0.35	Failure
220	20.52	14.06	26.23	25.38	9.86	0.37	Stable
221	21.9816	19.95665	22.005	19.981	180	0.1	Failure
222	18.46	12.004	0	29.998	6	0	Failure
223	20.45	16	15	30	36	0.25	Stable
224	21.1	33.5	28	40	31	0	Failure
225	22	20	36	45	30	0.29	Failure
226	17.6	10	16	21.8	9	0.4	Stable
227	31.3	68.6	37	47	270	0.25	Failure
228	23.4	15	38.5	30.3	45.2	0.28	Failure
229	16.472	11.55385	0	29.998	3.6	0	Failure
230	23.47	0	32	37	214	0.25	Failure
231	24.86	45.6	39.8	36.31	386.08	0.21	Stable
232	17.2	10	24.25	17.07	38	0.4	Stable
233	14.8	0	17	20	50	0	Failure
234	17.86	0	24.38	22.44	8.23	0.39	Stable
235	18.82	25	14.6	20.32	50	0.4	Failure
236	18.8292	10.35345	21.285	34.026	37	0.3	Failure
237	18.84	57.46	20	20	30.5	0.25	Stable
238	31.3	68.6	37	47	305	0.25	Stable
239	28.01	9.5	37.36	41.86	538.1	0.23	Stable
240	25	63	32	44.5	239	0.25	Stable
241	18.6	0	32	21.8	46	0.25	Stable
242	25.8	38.2	33	27	40	0	Stable
243	31.3	68	37	49	200.5	0.29	Failure
244	16	70	20	40	115	0.25	Failure
245	22	0	40	33	8	0.393	Stable
246	25	46	35	50	284	0.25	Stable
247	20.6	27.8	27	35	70	0	Failure
248	22	40	30	30	196	0	Stable
249	18.9712	30.01	35.01	34.98	11	0.2	Stable
250	26.2	43.8	38	35	68	0	Stable
251	17.9772	4.95165	30.015	19.981	8	0.3	Stable
252	22.4	28.9	24	28	35	0	Failure
253	25.6	39.8	36	30	32	0	Stable
254	19.36	19.8	38.49	43.41	48.88	0.43	Failure
255	20.41	24.9	13	22	10.7	0.35	Stable
256	23.5	10	27	26	190	0	Failure
257	17.4	20	24	18.43	51	0.4	Failure
258	17.6	10	8	21.8	9	0.4	Stable
259	22.3792	10.05335	35.01	29.998	10	0	Stable
260	21.7828	8.55285	31.995	27.984	12.8	0.49	Failure
261	19.63	11.97	20	22	12.19	0.405	Failure
262	25	48	40	45	330	0.25	Stable
263	25.8	39.4	33	25	45	0	Stable
264	27	40	35	47.1	292	0.25	Failure
265	12	0	30	35	8	0.25	Failure
266	22.3792	99.9333	45	44.997	15	0.25	Stable
267	16.5	11.49	0	30	3.66	0.25	Failure
268	25.8	34.7	33	30	50	0	Stable
269	26.62	0	31.78	42.72	51.48	0.4	Failure
270	24	0	40	33	8	0.3	Stable
271	18.84	0	20	20	7.62	0	Failure
272	18.7724	25.05835	19.98	29.998	50	0.2	Failure
273	22	21	23	30	257	0	Failure
274	23.2	9.5	39.69	39.34	10.49	0.44	Failure
275	21.78	0	34.2	35	7.13	0.32	Stable
276	14.8	0	17	20	50	0.25	Failure
277	31.3	68	37	47	213	0	Failure
278	21.8	32.7	27	50	50	0	Failure
279	21.8	28.8	26	35	99	0	Failure
280	26.2	42.8	37	30	37	0	Stable
281	22	10	35	30	10	0.29	Stable
282	19.6	21.8	29.5	37.8	40.3	0.25	Stable
283	18.6	0	32	26.5	46	0.25	Stable
284	27.3	10	39	41	511	0.29	Stable
285	28.07	35	38.93	44.54	361.51	0.24	Stable
286	19.63	11.97	20	22	12.2	0.41	Failure
287	27	50	40	42	407	0.29	Stable
288	21.73	9.21	30.6	33.06	19.78	0.29	Stable
289	27.3	14	31	41	110	0.29	Stable
290	26.69	50	26.6	50	170	0.25	Stable
291	26.5	35.4	32	30	21	0	Stable
292	26.5	41.8	36	42	54	0	Stable
293	18.7724	19.95665	9.99	25.016	50	0.3	Failure
294	29.7	38.09	32.92	45.48	410.4	0.26	Stable
295	26.2	42.3	36	23	36	0	Stable
296	20.6	16.28	26.5	30	40	0.25	Failure
297	20.9592	19.95665	40.005	40.015	12	0	Stable
298	20.6	28.5	27	40	65	0	Failure
299	17.29	0	37.22	44.55	42.3	0.28	Failure
300	12.34	0	25.92	46.82	8.08	0.43	Failure
301	27	37.5	35	37.8	320	0.29	Stable
302	24.9636	120.04	45	53	120	0	Stable
303	22.1	45.8	49.5	45.8	49.5	0.21	Stable
304	11.94	0	31.75	32.49	3.92	0.11	Stable
305	17.9772	45.015	25.02	25.016	14	0.3	Stable
306	20.6	32.4	26	30	42	0	Failure
307	18.8	8	26	21.8	40	0.4	Failure
308	31.3	68	37	47	360.5	0.25	Failure
309	26.83	13.98	35.46	43.5	96.14	0.23	Stable
310	21.2	0	35	23.75	150	0.25	Stable
311	22	0	36	45	50	0	Failure
312	17.9772	24.008	30.15	44.997	20	0.12	Failure
313	20	20	36	45	30	0.503	Failure
314	24.57	9.98	41.31	35.46	526.13	0.27	Stable
315	21.5	29.8	26	40	70	0	Failure
316	27.1	22	18.6	25.6	100	0.19	Failure
317	22	10	36	45	50	0.29	Failure
318	21.6	6.5	19	40	50	0	Failure
319	20.97	21.8	31.81	38.09	57.75	0.24	Failure
320	26.8	37.5	32	30	26	0	Stable
321	25.9576	150.05	45	49.979	200	0	Stable
322	19.9652	10.05335	28.98	34.026	6	0.3	Stable
323	22.54	29.4	20	24	210	0	Stable
324	26	42.4	37	38	55	0	Stable
325	20.41	24.9	13	22	10.67	0	Stable
326	21	20	24	21	565	0	Stable
327	31.3	68	37	49	200.5	0.25	Failure
328	20.6	32.4	26	35	55	0	Failure
329	16.05	11.49	0	30	3.66	0	Failure
330	25	46	36	44.5	299	0	Stable
331	19.43	11.16	0	32.34	5.35	0.36	Failure
332	20	30.3	25	45	53	0	Failure
333	21.9816	19.95665	36	44.997	50	0	Failure
334	27.3	31.5	29.703	41	135	0.293	Stable
335	21.5	15	29	41.5	123.6	0.36	Stable
336	20.8	14.8	21	30	40	0	Failure
337	25.8	43.3	37	30	33	0	Stable
338	20.41	33.52	11	16	45.72	0	Failure
339	27	40	35	47.1	292	0	Failure
340	24	40.8	35	35	50	0	Stable
341	22.4	100	45	45	15	0.25	Failure
342	25	63	32	46	300	0.25	Stable
343	18	24	30.2	45	20	0.12	Failure
344	26.81	60	28.8	59	108	0.25	Stable
345	28.35	44.97	33.49	43.16	413.42	0.25	Failure
346	19.0848	10.05335	9.99	25.016	50	0.4	Failure
347	27	27.3	29.1	35	150	0.26	Failure
348	31.3	68	37	8	305.5	0.25	Failure
349	25	48	40	49	330	0	Stable
350	18.8008	57.46915	19.98	19.981	30.6	0	Stable
351	27	32	33	42	301	0.25	Failure
352	25	46	35	46	393	0	Stable
353	18.84	0	20	20	7.6	0.45	Failure
354	20.3912	24.9083	13.005	21.995	10.6	0.35	Stable
355	26	15	45	50	200	0	Stable
356	31.3	58.8	35.5	47.5	438.5	0.25	Failure
357	18.6588	26.4088	14.985	34.98	8.2	0	Failure
358	21.1	10	30.34	30	20	0	Stable
359	25.8	41.2	35	30	40	0	Stable
360	21.4704	6.9023	30.015	31.005	76.8	0.38	Failure
361	23.47	0	32	37	214	0	Failure
362	20	0	20	20	8	0.35	Stable
363	23	20	20.3	46.2	40.3	0.25	Stable
364	31.3	58.8	35.5	47.5	502.7	0.25	Failure
365	26	39.4	36	25	30	0	Stable
366	27.3	10	39	40	480	0	Stable
367	21.8	27.6	25	35	60	0	Failure
368	21.4	28.8	20	50	52	0	Failure
369	19.9652	40.06335	30.015	29.998	15	0.3	Stable
370	20	8	20	10	10	0	Failure
371	23.8	31	38.7	47.5	23.5	0.31	Stable
372	26.6	42.4	37	25	52	0	Stable
373	28.4	39.16305	37.98	34.98	100	0	Stable
374	21.51	17.82	31.75	47.03	49.92	0.52	Failure
375	22	0	40	33	8	0.35	Failure
376	23	0	20	20	100	0.3	Failure
377	21.43	0	20	20	61	0	Failure
378	26.6	40.7	35	35	60	0	Stable
379	27.83	45.01	35.95	47.83	456.38	0.25	Stable
380	25	46	35	44	435	0.29	Stable
381	18.71	4.75	28.12	18.81	8.62	0.31	Stable
382	26.6	44.1	38	35	42	0	Stable
383	28.4	29.4098	35.01	34.98	100	0	Stable
384	19.028	11.7039	27.99	34.98	21	0.11	Failure
385	18.45	0	18.58	17.82	7.55	0.43	Failure
386	27	35	35	42	359	0.29	Stable
387	31.3	68.6	37	47.5	262.5	0.25	Failure
388	31.3	68	37	46	366	0.25	Failure
389	27	43	35	43	420	0.29	Failure
390	12	0	30	35	4	0.25	Stable
391	26.18	159	44.93	31.5	172.98	0.1	Failure
392	19.32	0	19.44	20.2	68.48	0.45	Failure
393	30	27.38	34.57	43.46	319.21	0.27	Failure
394	12	0	30	45	8	0	Failure
395	28.51	42.34	32.2	43.25	453.6	0.25	Stable
396	11.82	0	33.7	31.26	3.91	0.42	Stable
397	18.84	15.32	30	25	10.67	0	Stable
398	27	35.8	32	30	69	0	Stable
399	18	21	21.33	21.8	40	0.4	Failure
400	17.8	21.2	13.92	18.43	51	0.4	Stable
401	27.3	16.2	28	50	90.5	0.29	Stable
402	22.3	20.1	31	40.2	88	0.19	Stable
403	22.5	20	16	25	220	0	Stable
404	13.9728	12.004	26.01	29.998	88	0	Failure
405	25	46	35	46	432	0.29	Stable
406	20	30	36	45	50	0.29	Failure
407	23.2	31.2	23	30	33	0	Failure
408	25.4	33	33	20	35	0	Failure
409	26	150.05	45	50	200	0.25	Stable
410	19.9652	40.06335	40.005	40.015	10	0.2	Stable
411	20.3912	33.46115	10.98	16.006	45.8	0.2	Failure
412	28.44	39.23	38	35	100	0.25	Stable
413	21	10	30.343	30	30	0.29	Stable
414	22	29	15	18	400	0	Failure
415	27.8	27.8	27	41	236	0.1	Stable
416	26.5	42.9	38	34	36	0	Stable
417	18.8292	24.75825	21.285	29.203	37	0.5	Failure
418	21.9816	19.95665	22.005	19.981	180	0	Failure
419	18.8008	15.3051	30.015	25.016	10.6	0.38	Stable
420	21.83	8.62	32	28	12.8	0	Failure
421	22.85	8.46	38.12	25.67	11.34	0.56	Stable
422	18.5	25	0	30	6.003	0.29	Failure
423	27	38.4	33	25	22	0	Stable
424	24	41.5	36	30	51	0	Stable
425	21.43	0	20	20	61	0.5	Failure
426	26	150	45	30	230	0.29	Stable
427	18.5	12	0	30	6.003	0.29	Failure
428	22.3792	10.05335	35.01	44.997	10	0.4	Failure
429	20.5616	16.2054	26.505	29.998	40	0	Failure
430	31	68	37	46	366	0.25	Failure
431	21.3568	10.05335	30.33	29.998	20	0	Stable
432	25	46	35	50	284	0	Stable
433	27	32	33	42.2	239	0.29	Stable
434	25.6	36.8	34	35	60	0	Stable
435	20	0	36	45	50	0.5	Failure
436	19.0848	10.05335	19.98	29.998	50	0.4	Failure
437	33.16	68.54	41.11	51.98	188.15	0.44	Failure
438	21.2	0	35	18.43	73	0.25	Stable
439	20.6	26.31	22	25	35	0	Failure
440	18.46	25.05835	0	29.998	6	0	Failure
441	22.3	0	40	26.5	78	0.25	Stable
442	12	0	30	35	4	0.29	Stable
443	18.12	10.57	30.84	32.45	21.77	0.11	Failure
444	19.6	29.6	23	40	58	0	Failure
445	27	27.3	29.1	37	184	0.22	Failure
446	25	55	36	45	299	0.25	Stable
447	22.5	18	20	20	290	0	Stable
448	18.8008	14.4048	25.02	19.981	30.6	0.45	Failure
449	12	0	30	45	4	0	Stable
450	23.47	0	32	37	214	0	Stable
451	20.41	33.52	11	16	10.67	0.35	Stable
452	25.4	33	33	20	35	0	Stable
453	27.3	31.5	30	41	135	0.25	Stable
454	21.4	10	30	30	20	0.25	Stable
455	18.66	8.8	15	35	8.2	0	Failure
456	28.4	9.8	35	35	100	0	Stable
457	25.96	50	45	50	200	0	Stable
458	18.46	8.35	0	30	6	0	Failure
459	21.36	3.35	30	30	20	0	Stable
460	15.99	23.35	20	40	115	0	Failure
461	20.39	8.3	13	22	10.6	0.35	Stable
462	19.6	4	20	22	12.2	0.41	Failure
463	20.39	11.15	11	16	45.8	0.2	Failure
464	19.03	3.9	28	35	21	0.11	Failure
465	17.98	1.65	30	20	8	0.3	Stable
466	20.96	6.65	40	40	12	0	Stable
467	20.96	11.65	28	40	12	0.5	Stable
468	19.97	3.35	29	34	6	0.3	Stable
469	18.77	10	10	25	50	0.1	Stable
470	18.77	10	20	30	50	0.1	Stable
471	18.77	8.35	20	30	50	0.2	Failure
472	20.56	5.4	27	30	40	0	Failure
473	16.47	3.85	0	30	3.6	0	Failure
474	18.8	4.8	25	20	30.6	0	Stable
475	18.8	19.15	20	20	30.6	0	Stable
476	28.4	13.05	38	35	100	0	Stable
477	24.96	40	45	53	120	0	Stable
478	18.46	4	0	30	6	0	Failure
479	22.38	3.35	35	30	10	0	Stable
480	21.98	6.65	36	45	50	0	Failure
481	18.8	5.1	30	25	10.6	0.38	Stable
482	18.8	4.8	25	31	76.8	0.38	Failure
483	21.47	2.3	30	30	88	0.45	Failure
484	13.97	4	26	45	20	0.12	Failure
485	17.98	8	30	45	15	0.25	Failure
486	22.38	33.3	45	45	10	0.4	Stable
487	22.38	3.35	35	45	50	0.25	Failure
488	19.97	6.65	36	45	50	0.25	Failure
489	19.97	6.65	36	45	50	0.5	Failure
490	20.96	15	25	49	12	0.3	Stable
491	20.96	10	35	40	12	0.4	Stable
492	19.97	13.35	30	30	15	0.3	Stable
493	17.98	15	25	25	14	0.3	Stable
494	18.97	10	35	35	11	0.2	Stable
495	19.97	13.35	40	40	10	0.2	Stable
496	18.83	8.25	21	21	37	0.5	Stable
497	18.83	3.45	21	34	37	0.3	Failure
498	18.77	8.35	10	25	50	0.2	Failure
499	18.77	6.65	10	25	50	0.3	Failure
500	19.08	3.35	10	25	50	0.4	Failure
501	18.77	6.65	20	30	50	0.3	Failure
502	19.08	3.35	20	30	50	0.4	Failure
503	21.98	6.65	22	20	180	0	Failure
504	21.98	6.65	22	20	180	0.1	Failure
505	20	20	36	45	50	0	Failure
506	27	27.3	29.1	21	565	0.26	Failure
507	27	27.3	29.1	35	150	0.22	Failure
508	27	27.3	29.1	37	184	0.3	Failure
509	0.657	0.176	0.333	0.66	0.041	0	Failure
510	1	0.196	0.778	0.66	0.5	0	Stable
511	0.914	1	1	0.943	1	0	Stable
512	0.65	0.167	0	0.566	0.03	0	Failure
513	0.752	0.067	0.674	0.566	0.1	0	Stable
514	0.563	0.467	0.444	0.755	0.575	0	Failure
515	0.718	0.166	0.289	0.415	0.053	0.7	Stable
516	0.69	0.08	0.444	0.415	0.061	0.81	Failure
517	0.767	0.057	0.711	0.528	0.064	0.98	Failure
518	0.718	0.223	0.244	0.302	0.229	0.4	Failure
519	0.67	0.078	0.622	0.66	0.105	0.22	Failure
520	0.633	0.033	0.667	0.377	0.04	0.6	Stable
521	0.738	0.133	0.889	0.755	0.06	0	Stable
522	0.738	0.233	0.622	0.755	0.06	1	Stable
523	0.703	0.067	0.644	0.642	0.03	0.6	Stable
524	0.661	0.2	0.222	0.472	0.25	0.2	Stable
525	0.661	0.2	0.444	0.566	0.25	0.2	Stable
526	0.661	0.167	0.444	0.566	0.25	0.4	Failure
527	0.724	0.108	0.589	0.566	0.2	0	Failure
528	0.58	0.077	0	0.566	0.018	0	Failure
529	0.662	0.096	0.556	0.377	0.153	0	Stable
530	0.662	0.383	0.444	0.377	0.153	0	Stable
531	1	0.261	0.844	0.66	0.5	0	Stable
532	0.492	0.08	0.578	0.566	0.44	0	Failure
533	0.879	0.8	1	1	0.6	0	Stable
534	0.65	0.08	0	0.566	0.03	0	Failure
535	0.788	0.067	0.778	0.566	0.05	0	Stable
536	0.774	0.133	0.8	0.849	0.25	0	Failure
537	0.662	0.102	0.667	0.472	0.053	0.76	Stable
538	0.662	0.096	0.556	0.377	0.153	0.9	Failure
539	0.756	0.046	0.667	0.585	0.384	0.76	Failure
540	0.492	0.08	0.578	0.566	0.44	0.9	Failure
541	0.633	0.16	0.67	0.849	0.1	0.24	Failure
542	0.788	0.666	1	0.849	0.075	0.5	Stable
543	0.788	0.067	0.778	0.849	0.05	0.8	Failure
544	0.703	0.133	0.8	0.849	0.25	0.5	Failure
545	0.703	0.133	0.8	0.849	0.25	1	Failure
546	0.738	0.3	0.556	0.925	0.06	0.6	Stable
547	0.738	0.2	0.778	0.755	0.06	0.8	Stable
548	0.703	0.267	0.667	0.566	0.075	0.6	Stable
549	0.633	0.3	0.556	0.472	0.07	0.6	Stable
550	0.668	0.2	0.778	0.66	0.055	0.4	Stable
551	0.703	0.267	0.889	0.755	0.05	0.4	Stable
552	0.633	0.165	0.473	0.551	0.185	1	Failure
553	0.633	0.069	0.473	0.642	0.185	0.6	Failure
554	0.661	0.167	0.222	0.472	0.25	0.4	Failure
555	0.661	0.133	0.222	0.472	0.25	0.6	Failure
556	0.672	0.067	0.222	0.472	0.25	0.8	Failure
557	0.661	0.133	0.444	0.566	0.25	0.6	Failure
558	0.672	0.067	0.444	0.566	0.25	0.8	Failure
559	0.774	0.133	0.489	0.377	0.9	0	Failure
560	0.774	0.133	0.489	0.377	0.9	0.2	Failure
561	17.6	39.5	30.2	50	38	0.04	Stable
562	17.3	39	30	50	35	0.04	Stable
563	17.8	38.7	30.5	60	26	0	Stable
564	17.9	39	31.2	55	25	0.15	Stable
565	17.3	39	30	50	26	0.2	Stable
566	17.3	37.9	30	45	29	0.37	Stable
567	17.5	38.5	29	50	33	0.2	Stable
568	17.5	39.2	29.7	55	31	0	Stable
569	17.8	39.8	31.3	45	32	0.34	Stable
570	17.3	39	30	48	30	0.03	Stable
571	18.3	57.2	38.6	38	31	0.64	Stable
572	17.4	5	43.5	58	29	0.05	Failure
573	17.8	14	44.2	65	31	0.07	Failure
574	17.4	0	43.7	60	26	0.4	Failure
575	19.8	57.5	41.3	62	23	0.19	Stable
576	20.5	6.5	12.5	42	70	0	Failure
577	21.4	7.1	16.7	44	70	1	Failure
578	21.5	9.5	11.5	40	75	0	Failure
579	20.6	6.7	9.4	45	30	0	Failure
580	20.9	9.7	18.5	39	38	1	Failure
581	21.4	9.4	21.8	30	106	1	Failure
582	19.9	6.8	19.4	30	80	1	Failure
583	20.2	14.9	18.5	40	70	1	Failure
584	19	9	15.2	45	27	0	Failure
585	19.7	16.4	21.4	30	55	1	Failure
586	21.2	7.8	22.4	45	25	1	Failure
587	19.9	7.4	15.6	44	30	1	Failure
588	19.9	7.1	21.2	30	55	0	Failure
589	22.2	10.7	25.2	35	45	1	Failure
590	21.8	7.2	17.8	40	34	1	Failure
591	21.8	7.2	17.8	42	41	1	Failure
592	21.96	34.77	14.15	28	60	0	Stable
593	21.96	34.77	14.15	24	115	0	Stable
594	22.93	32.33	19.73	30	50	1	Stable
595	22.15	19.47	13.29	28	110	1	Stable
596	23.4	20	9	36.5	50	0	Stable
597	21.8	18.05	9.72	30	40	0	Failure
598	23.98	32.77	17.28	40	100	0	Failure
599	20.57	24.8	15.53	40	50	1	Stable
600	21.2	24.88	17.29	44	52	0	Failure
601	22.15	5	19	45	40	1	Failure
602	21.8	18.05	9.72	35	40	0	Failure
603	23.75	36.78	22.63	42	43	1	Failure
604	20.98	23.59	20	45	65	0	Failure
605	22.6	24.06	14.04	26	190	1	Stable
606	22.29	27.54	10.1	40	70	0	Stable
607	22.1	24.67	16.2	40	70	1	Stable
608	20.25	32.4	11.99	45	36	1	Failure
609	20.8	15.57	8.74	29.7	35	1	Failure
610	21.17	15.44	16	33	32	1	Failure
611	22.94	33.77	23.29	27	170	1	Stable
612	22.95	46.49	25.11	30	42	1	Stable
613	21.92	19.4	15.5	35	80	1	Failure
614	21.42	28.9	16.2	40	30	1	Stable
615	20.8	40.25	19.39	45	123	1	Failure
616	20.1	34.61	24.69	22	94	0	Stable
617	19.19	19.69	17.68	34	43	1	Failure
618	19.18	12.8	9.45	45	20	0	Failure
619	17.8	22.2	6.05	40	51.6	1	Failure
620	19.6	15.53	15.88	35	97	1	Failure
621	19.81	33.75	19.46	20	120	1	Stable
622	19.81	19.97	11.08	35	35	0	Failure
623	19.7	17	9.38	45	20	1	Failure
624	20.2	21.2	19.89	35	62	1	Failure
625	17.96	24.01	28	40	60	1	Failure
626	25	55	36	44.5	299	0.25	Stable
627	21.98	19.96	22.01	19.98	180	0.01	Failure
